# Vaccination and hybrid immunity is associated with SARS-CoV-2 protection but not shedding duration in rural Guatemalan households

**DOI:** 10.21203/rs.3.rs-9348647/v1

**Published:** 2026-05-21

**Authors:** Bradley S. Barrett, Molly M. Lamb, Kejun Guo, Diva M. Calvimontes Barrientos, Neudy Rojop, Claire Bradley, Hashem Anabtawi, Kareen Arias, Claudia Paiz, Julio del Cid-Villatoro, Edwin J. Asturias, Mario L. Santiago, Daniel Olson

**Affiliations:** 1. Department of Medicine, Division of Infectious Diseases, University of Colorado School of Medicine, Aurora, CO, USA; 2. Department of Epidemiology and Center for Global Health, Colorado School of Public Health, 13199 East Montview Blvd, Aurora, CO, USA; 3. Center for Human Development, Fundacion para la Salud Integral de los Guatemaltecos, FSIG, Retalhuleu, Guatemala; 4. La Comisión Presidencial de Atención a la Emergencia COVID-19 (Coprecovid), Guatemala City, Guatemala; 5. Department of Pediatrics, Division of Infectious Disease, University of Colorado School of Medicine, 13123 E. 16th Ave., Aurora, CO, USA;

## Abstract

**Background:**

Essential agricultural workers in Central America and their households faced a high force of infection during the COVID-19 pandemic. Here, we evaluated if pre-existing seropositivity and vaccination (hybrid immunity) influenced SARS-CoV-2 infection risk and duration of shedding in an agricultural community in rural Guatemala.

**Methods:**

Individuals from 70 households were enrolled in a community cohort. From September 2021 to December 2022, all participants reported symptoms twice-weekly and provided saliva weekly for SARS-CoV-2 RNA testing by reverse transcriptase polymerase chain reaction (RT-PCR). Upon SARS-CoV-2 detection, participants submitted saliva three times per week for four weeks, which was tested by RT-PCR, to assess duration of shedding. We selected 119 SARS-CoV-2 PCR-positive individuals and 101 month-matched controls to measure nucleocapsid IgG and Omicron neutralizing antibody (NAb) titers.

**Results:**

Compared to uninfected controls, SARS-CoV-2 cases were more frequently female (60.5% vs 48.5%, p=0.08), had fewer vaccine doses (p=0.07), and were less likely to have hybrid immunity (p=0.08). Compared to controls, SARS-CoV-2 cases had lower median titers of nucleocapsid IgG (cutoff index [COI]=9.6 vs 23.6, p=0.12) and Omicron NAb (28.4 vs 50.4, p=0.15). SARS-CoV-2 cases also had lower NAb than controls among those vaccinated with 1 (16.1 vs 182.7, p=0.01), 2 (11.9 vs 87.0, p=0.04), or 3 doses (13.4 vs 232.5, p=0.004), and those considered up-to-date on vaccination (13.1 vs 155.6, p=0.0001). Nucleocapsid IgG and Omicron NAb were similar between individuals with short (≤7 days) vs long (>7 days) viral shedding durations.

**Conclusions:**

In a household cohort of Guatemalan agricultural workers, cumulative vaccine doses associated with lower odds of infection. Among those previously vaccinated, lower titers of nucleocapsid IgG and Omicron NAb were associated with elevated infection risk, highlighting the importance of hybrid immunity in COVID-19 protection in this population.

## BACKGROUND

Essential workers in the agricultural sector and their household contacts may be at increased risk of SARS-CoV-2 exposure and transmission, due to occupational exposures and crowded living conditions (1, 2). Defining correlates of protective immunity against SARS-CoV-2 infection and COVID-19 disease in agricultural worker populations and their communities is important given their high risk of SARS-CoV-2 exposure and their importance in global food security. Though many studies have evaluated serologic immunity as a correlate of protection in diverse populations (3–12), primarily against COVID-19 disease, few studies have focused on agricultural communities, especially in low- and middle-income country (LMIC) settings with differing risks of exposure and disease severity (2, 13–15). Correlates of protective immunity in this highly exposed, essential worker population may be different than other risk groups and could be used to predict population-level susceptibility to future infection or as vaccine immunobridging studies (9, 16).

We previously characterized significant clinical and economic consequences of COVID-19 in a cohort of essential agricultural workers in rural southwest Guatemala (17, 18). Subsequently, we demonstrated a high force of infection in the households of a subset of those workers enrolled in the related “AGRIcultural worker COVID-19 Asymptomatic and SymptomAtic transmission in the Home and Workplace (AGRI-CASA) Study” (19). The AGRI-CASA study included extensive monitoring of household contacts of SARS-CoV-2 index cases that could provide insights on factors influencing human-to-human SARS-CoV-2 transmission. Here, we aimed to evaluate the protective effect of pre-existing serologic immunity and vaccination on subsequent SARS-CoV-2 infection and shedding among household participants enrolled in the AGRI-CASA study, encompassing successive infection waves due to Omicron and its subvariants.

## METHODS

The present study was a nested case-control analysis performed among a subset of participants who had enrolled in the parent AGRI-CASA cohort study (19). The AGRI-CASA study was conducted in two rural, resource-limited communities in the coastal lowlands of southwest Guatemala. (17, 20, 21); rolling enrollment included 70 households from the two communities. Households that ended participation were replaced to maintain the 70-household cohort. AGRI-CASA enrollment criteria included having ≥1 household member employed at a large agribusiness who participated in the separate AGRI study, residing within the two study communities, and at least 75% of household members consenting to participate.

### Data Collection and Routine Surveillance

Following informed consent and assent, as appropriate, AGRI-CASA study nurses collected standardized demographic, clinical, and epidemiologic data, including household-level (number of adults/children, environmental data) and individual-level (sex, vaccination status, vaccination date, and self-reported medical conditions) characteristics. All enrolled participants were screened twice weekly for COVID-19-like Illness (CLI) defined as ≥1 of the following symptoms for ≥48 hours: fever/chills, cough, shortness of breath, fatigue, muscle/body ache, headache, loss of taste/smell, sore throat, congestion, nausea, vomiting, or diarrhea (22, 23).

### Specimen collection and testing

Each week, regardless of symptom screening results, 5 ml of saliva was collected for future virologic testing. In addition to weekly saliva collection, participants with symptoms meeting CLI criteria or with confirmed community or household exposure to SARS-CoV-2 in the past three days received an additional visit for saliva collection.

Saliva samples from individuals with CLI or exposure were tested for SARS-CoV-2 by PCR within 24 hours. Subjects with negative PCR results returned to routine surveillance. For households with a SARS-CoV-2-positive swab, all enrolled household contacts provided detailed clinical and epidemiological data through six weeks. Household contacts of PCR-positive individuals also provided blood specimens, and saliva three times per week for four weeks, regardless of their symptom or laboratory results (19). Following the 6-week visit, households returned to routine surveillance.

### SARS-CoV-2 PCR Testing

All saliva specimens were transported on ice, processed, and stored at −80 °C within 24 hours, as previously described (19). Specimens were processed according to the saliva-direct protocol (24), mixing 200 μl of saliva with 400 μl of 0.9% saline then added to the Roche Cobas^®^ assay tube. Saliva specimens from symptomatic individuals identified during routine surveillance were tested off-label within 24 hours (25) using the Roche point-of-care (POC) Cobas^®^ Liat real-time PCR (RT-PCR) Influenza A/B & RSV and SARS-CoV-2 kits (26). POC PCR results were shared with participants within 24 hours. Specimens from households collected after the incident detection of SARS-CoV-2, influenza, or RSV were tested at a later date by RT-PCR testing (CDC Protocol (27)) at Universidad del Valle de Guatemala (UVG) or digital droplet PCR at Colorado State University (Fort Collins, CO) (28). Retrospectively, saliva collected from all household contacts during routine surveillance in the two weeks preceding the incident household viral detection were also tested. POC and batched RT-PCR results were shared weekly with the Guatemala Ministry of Health.

### Selection of Cases and Controls for Sub-Study

**Figure 1:**
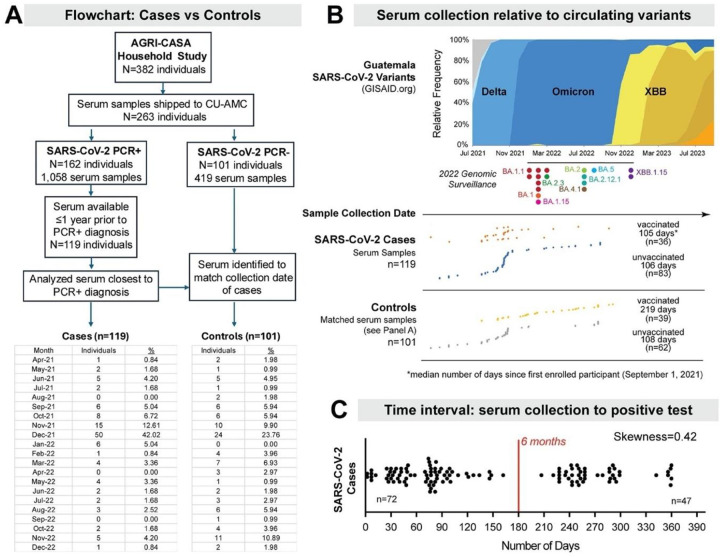
Evaluation of Pre-Existing Serological Immunity in AGRI-CASA. (A) Flowchart for selection of SARS-CoV-2 cases and controls. Serum samples from the 2 cohorts were matched for collection date to capture similar circulating variants. (B) Circulating variants in Guatemala relative to date of serum sample collection. Each dot corresponds to serum samples from SARS-CoV-2 cases and month-matched controls, subdivided based on vaccination status. Each dot in the Genomic Surveillance inset corresponds to a full-length SARS-CoV-2 sequence from one individual collected during specific months in 2022. Sampling occurred primarily during waves due to Omicron (BA.1) and its subvariants based on deposited SARS-CoV-2 sequences from AGRI-CASA and sequences from Guatemala deposited in GISAID.org. The median number of days for sample collection of each group was noted, relative to the first reported subject enrolled (Sep 1, 2021), showing vaccinated controls were sampled about 3.7 months later than the other groups. (C) Distribution of time intervals between sample collection and positive SARS-CoV-2 test for cases, showing a bimodal distribution (skewness=0.42).

“Cases” were defined as individuals of any age enrolled in the AGRI-CASA study with PCR-confirmed SARS-CoV-2 infection and a serum sample available at the University of Colorado-Anschutz Medical Campus (CU-AMC) collected during the 12 months preceding infection. “Controls” were individuals without SARS-CoV-2-confirmed infection who had a prior serum sample available at CU-AMC. Control serum samples were selected to match case serum samples by month of collection; for case:control pairs that did not have month-matched serum samples, control serum samples were selected so the median overall collection dates were similar between cases and controls (Figure 1A).

### SARS-CoV-2 Serologic Testing

At the Santiago Laboratory at CU-AMC, serum was tested for anti-nucleocapsid IgG, reflective of prior infection, using the Roche Diagnostics Elecsys^®^ anti-SARS-CoV-2 immunoassay, as previously described (18). All specimens were run in triplicate on the Roche Elecsys^®^ e801 per the manufacturer’s instructions and reported as cut-off index (COI) value. The assay was previously reported to have a sensitivity of 95% and specificity of 99.8% at >14 days post-PCR positive SARS-CoV-2 patient samples (29).

To measure SARS-CoV-2 neutralizing antibodies (NAbs), we utilized a lentivirus-based assay as previously described (18, 30) (**Supplemental Figure 1A**). Briefly, HIV-1Δenv with a nanoluciferase reporter (Dr. Paul Bieniasz, Rockefeller University) was pseudotyped with codon-optimized SARS-CoV-2 Spike based on the Omicron strain (B.1.1.529 or BA.1; Invivogen catalog number p1-spike-v11) in 293T cells (31). Pseudovirions (~3–4×10^5^ relative light units, RLU) were co-incubated with serial 5-fold dilutions of serum (from 1:10 to 1:31250) in 100 μl complete media (F-12 Ham’s Medium with 10% FBS) containing 10,000 A549-ACE2 cells for 1 h at 37°C (32). The virus-serum-cell mixture was then plated into 96-well white plates, lysed after 48 h with Nano-Glo substrate (Promega) and RLUs were measured in a Victor X5 luminometer (Perkin Elmer). 50% inhibitory concentrations (IC50) were calculated using a two-phase decay equation (GraphPad Prism; **Supplemental Figure 1B**).

### COVID-19 Vaccination

Guatemala first received COVID-19 vaccine through the COVAX mechanism on March 11, 2021, with Pfizer and Moderna vaccines comprising the majority (19). COVID-19 vaccination dates, doses, and manufacturer data were collected directly from the Guatemalan Ministry of Public Health centralized database. Individuals with no evidence of vaccination were considered unvaccinated, and number of doses (up to 4 doses) was documented. We also categorized vaccination status based on whether the most recent vaccine dose (at or after completion of the primary series) was received during the past 12 months. “Hybrid immunity” was determined by inferring SARS-CoV-2 infection from anti-N IgG positivity (see below) and ≥1 vaccine dose. We carried out a separate analysis excluding individuals who had a vaccine dose between the serum sample collection and the symptomatic visit.

### Index and Secondary Case Definition

An index or co-index case was defined as the first person(s) who tested positive for SARS-CoV2 within a household. A secondary case was defined as a household contact who tested positive during 1–14 days after the index case date. For analysis purposes, a tertiary case was defined as any household contact who tested positive 14–42 days after the index case. Forty-two days after the incident index case infection, the household was considered clear of infection and susceptible to subsequent infection. Number of days positive for SARS-CoV-2 was calculated as days between the first and last day of PCR-positive test results within the 42-day window.

### Statistical analysis

Three major questions were assessed: (1) whether SARS-CoV-2 infection status was associated with prior vaccination; (2) whether SARS-CoV-2 infection status was associated with prior serum antibody positivity/titers in the context of vaccination; and (3) whether SARS-CoV-2 viral shedding duration was associated with preceding serum antibody positivity/titer and vaccination. For continuous variables, medians and interquartile ranges (IQRs) were calculated for individual-level characteristics stratified by prior infection status based on enrollment serum testing; means were calculated and compared via t-test, and categorical demographic variables were compared using chi-square test or Fisher’s exact test, as appropriate. Analysis of cut-off index (COI) values for SARS-CoV-2 anti-nucleocapsid (anti-N) IgG and IC_50_ for SARS-CoV-2 neutralizing antibody titer was completed using Prism software. Analysis was completed using two-way ANOVA with post-test analysis, t-test, and correlation analysis. A COI value > 1 was considered positive for anti-N IgG, as specified by the manufacturer. Comparisons of anti-N IgG positivity rates between 2 groups were evaluated using a 2-tailed Chi-Square test in Prism (version 10.2.3). Simple linear regression of log-transformed values was used to determine if there was a correlation between Omicron NAb and anti-N IgG titers, as well as the relationship between these antibody markers and the number of vaccinations. To determine differences in IgG and NAb titers by demographic variables, median titers were calculated by group or subgroup, and compared using the Wilcoxon two-sample test. Statistical analyses were conducted using SAS v.9.2 (Cary, NC).

### Data availability

The data underlying this article will be made available on request to the corresponding authors.

### Ethical Oversight

The study was carried out in accordance with the Declaration of Helsinki. The study was approved by the Colorado Multiple Institutional Review Board (COMIRB protocol #21–2551), the UVG Ethics Committee (#225-01-2021), and CDC (#212551) as human subjects research. The local SW Trifinio Community Advisory Board for Research agreed to the study. All study participants provided written informed consent.

## RESULTS

Between September 28, 2021, and December 21, 2022, 382 participants were enrolled in the AGRI-CASA cohort. Of these individuals, 263 participants had serum samples available at CU-AMC. Of these individuals, we identified 119 SARS-CoV-2 cases (Figure 1A), with the majority (n=95, 79.8%) of these serum samples collected during the start of the Omicron (BA.1) wave in Guatemala (November 2021) and extending towards the XBB subvariant wave, based on deposited sequences in GISAID.org and genomic surveillance of within the cohort (Figure 1B). An additional 29 SARS-CoV-2-positive individuals did not have a serum sample available prior to infection, and 14 had a sample available >12 months preceding their infection. Of the 119 SARS-CoV-2 cases, 38 (31.9%) met the COVID-like illness (CLI) case definition on the date of their first SARS-CoV-2 PCR test or during the shedding period; 81 (68.1%) were considered subclinical (did not meet the CLI case definition); 82 (68.9%) individuals were household contacts of index cases. The median duration of viral shedding was 7 days but varied widely from 1 to 29 days.

We identified 101 controls. Eighteen cases did not have sufficient date-matched controls. Using the first enrolled participant (September 1, 2021) as a reference point (19), the median serum collection date for cases and controls were similar (105 versus 117 days). SARS-CoV-2 cases were more frequently female (60.5% vs 48.5%, p=0.08) but were otherwise similar to controls by age and presence of pre-existing medical conditions (Table 1). Among cases, there were no differences in demographic characteristics between individuals who shed SARS-CoV-2 for ≤7 days (n=45) vs >7 days (n=37) (**Supplemental Table 1**).

To determine if SARS-CoV-2 cases differed from controls in terms of vaccination status, we first confirmed that the duration between vaccination and serum collection between SARS-CoV-2 cases and controls was not significantly different (123 days vs 97 days, p=0.47; Table 1). When we compared subgroups, we found that serum samples from vaccinated controls (median 218 days following first enrolled participant) were collected a median 3.7 months later than vaccinated cases (104 days), unvaccinated controls (108 days), and unvaccinated cases (105 days) (p<0.05), and thus were more frequently collected during Omicron subvariant circulation (after December 1, 2021; 84.6% vs 63.9%, 56.5%, and 68.7%, respectively; p=0.018). Despite this difference in timing of sample collections, there were no overall differences in baseline NAb titer when comparing unvaccinated cases to controls during this time period from January to April, 2022 (IC_50_=52.4 vs 31.3, p=0.93, n=11).

The proportion of individuals who received ≥1 COVID-19 vaccine dose was similar between SARS-CoV-2 cases and controls (46.2% vs 50.5%; p=0.53). As outlined in Table 1, cases and controls did have differences in the number of vaccine doses and in hybrid immunity status. Among SARS-CoV-2 cases, after removing 29 individuals who had a COVID-19 vaccine dose between their serum sample collection and their symptomatic visit, these differences persisted (**Supplemental Table 2**).

**Figure 2:**
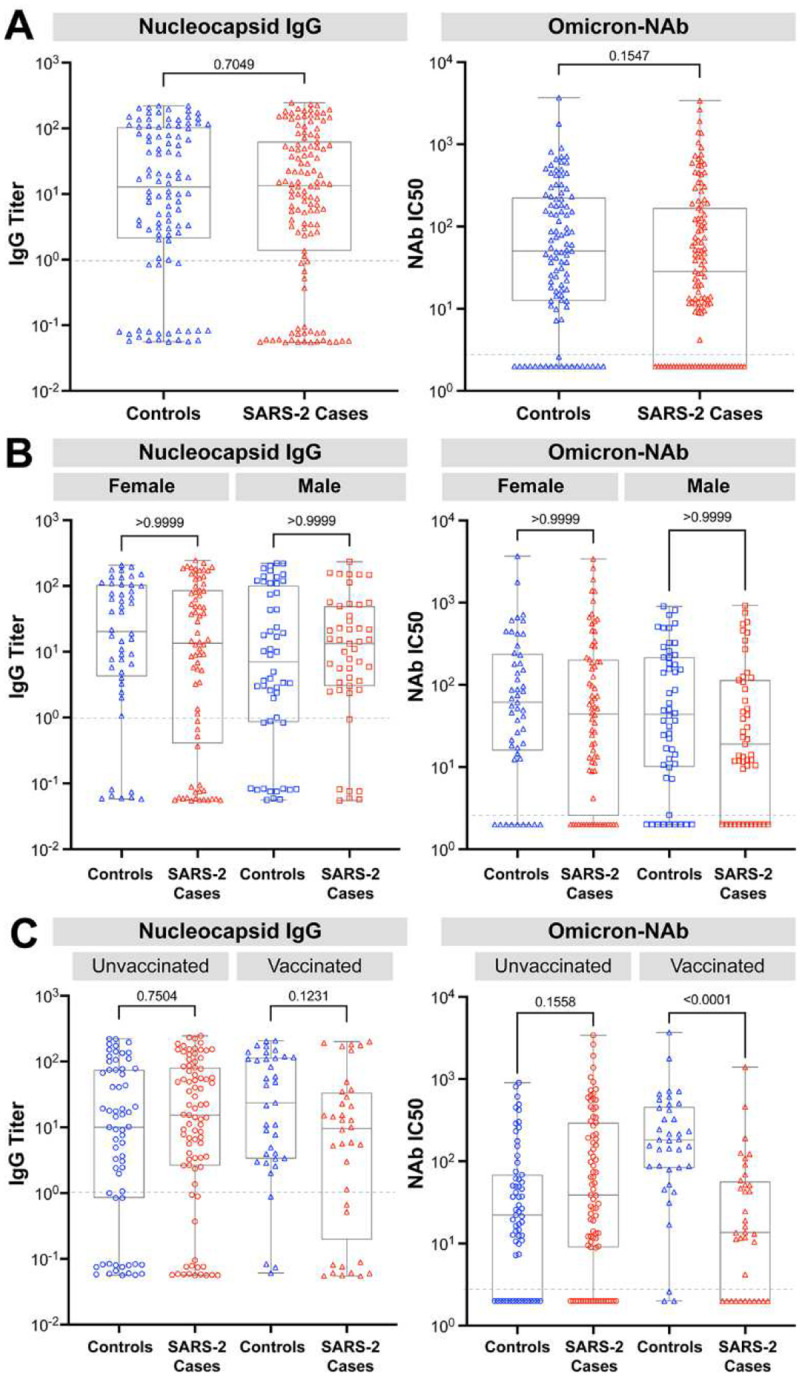
Antibody titers in serum samples prior to documented SARS-CoV-2 infection in AGRI-CASA. Serum samples were evaluated for nucleocapsid (N) IgG titers (left panels) and Omicron neutralizing antibody (NAb) titers (right panels). Each dot corresponds to a single individual, analyzed (A) without stratification, and (B,C) stratified according to (B) Sex and (C) Vaccination status. Bars and lines correspond to median ± IQR. Data were analyzed using One-Way ANOVA (Kruskal-Wallis test) followed by a Dunn’s Multiple Comparisons test, with p-values indicated.

SARS-CoV-2 cases and controls had similar anti-N IgG positivity rates (76.5% vs 77.2%, p=0.79) and anti-N IgG titers (median IgG COI =13.3 vs 12.8, p=0.7; Figure 2A). We next measured NAbs against the Omicron (BA.1) variant and detected no statistically significant difference in SARS-CoV-2 cases versus controls (IC_50_ = 28.4 vs 50.4, p=0.15) in the 12 months preceding their infection (Table 2, Figure 2A). Among SARS-CoV-2 cases (n=119), we found no evidence for differences in median Omicron NAb titer between those infected during the 6 months after serum collection (n=72) and those infected 6–12 months after serum collection (n=47) (IC_50_ = 28.8 vs 28.4, *p*=0.33; Figure 1C). Symptomatic (CLI) SARS-CoV-2 cases had no significant difference in median IgG (43.2 vs 21.8, p=0.33) or median NAb titer (15.4 vs 10.0, p=0.26) preceding their infection compared to asymptomatic cases.

We evaluated if there were differences in antibody titers based on vaccination status. As outlined in Table 2, among unvaccinated individuals, there was no difference in Omicron NAb titer between cases and controls (IC_50_=0.11). However, among vaccinated individuals (1, 2, or 3 doses), controls had significantly greater NAb titers, including among those considered “up-to-date” on vaccination (155.6 vs 12.1, p=0.0001); anti-N IgG was similar between cases and controls among those considered up-to-date on vaccination (COI=10.1 vs 16.3, p=0.17). After removing the 29 cases that had a COVID-19 vaccine dose between their serum sample and their symptomatic visit, these differences persisted (**Supplemental Table 3**).

Among controls, vaccination correlated with enrollment Omicron NAb titers (indicating vaccine- or infection-induced immunity; R^2^=0.20, p<0.0001) but not with anti-nucleocapsid IgG titers (indicating infection-induced immunity; R^2^=0.03, p=0.07) (**Supplemental Figure 2A).** Among unvaccinated individuals, SARS-CoV-2 cases and controls had similar median anti-N IgG (COI=15.4 vs 10.0, p=0.75) and Omicron NAb titers (IC_50_=38.9 vs 24.5, *p*= 0.11). Both groups also had similar anti-N IgG positivity rates (79.5% vs 71.4%, *p*=0.26). However, among vaccinated individuals, SARS-CoV-2 cases exhibited 13.3-fold lower Omicron NAb titers than controls (IC_50_ = 13.6 vs 181.2, p<0.0001) (Figure 2C; **Supplemental Figure 1B**). This difference persisted when stratified to individuals based on 1 (11.3-fold, p=0.03), 2 (7.3-fold, p=0.20) and 3 vaccine doses (17.4-fold, p=0.0083) (**Supplemental Figure 2C**).

**Figure 3.**
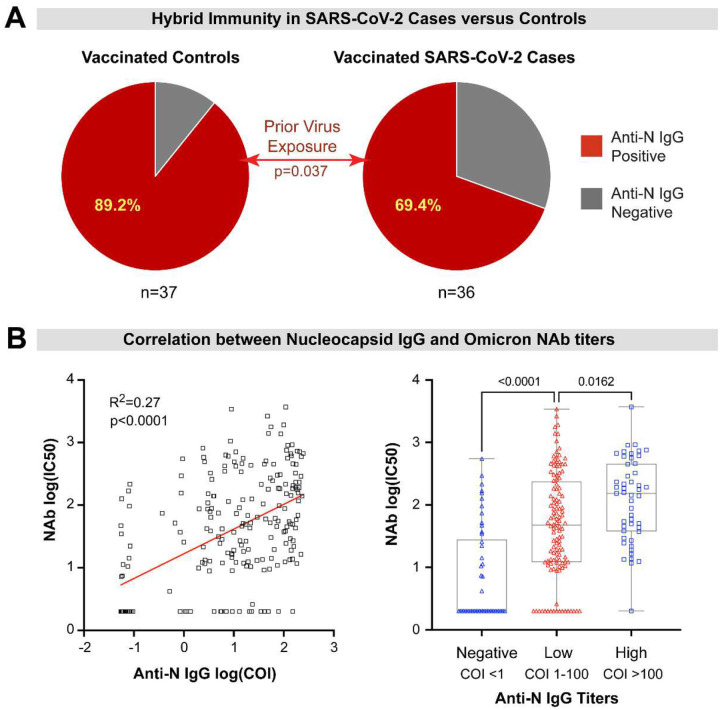
Evaluation of Hybrid Immunity in the AGRI-CASA study. Individuals who were previously vaccinated were evaluated for anti-N positivity, which is an indicator of prior virus exposure. (A) The proportion of anti-N IgG+ individuals were compared between vaccinated controls and SARS-CoV-2 cases by Chi-Square test. (B) Linear correlation between log-transformed anti-N IgG and Omicron NAb titers for the entire cohort which included both vaccinated and unvaccinated individuals (left panel). Omicron NAb titers were compared between samples with negative, low and high anti-N IgG titers. Median ± IQR values are shown and evaluated using the Kruskall-Wallis test and Dunn’s Multiple Comparison post-hoc test, with p-values shown.

The median anti-N IgG titer among SARS-CoV-2 cases was 2.4-fold lower than controls (COI =9.6 vs 23.6), although this difference did not reach statistical significance (p=0.12). We also did not observe a statistical difference in anti-N IgG titers when stratified based on vaccination doses (**Supplemental Figure 2B**). Anti-N IgG positivity rates were significantly lower in vaccinated SARS-CoV-2 cases compared to vaccinated controls (69.4% vs 89.2%, *p*=0.037; Figure 3A). When restricted just to those who received a vaccination within 6 months (n=55), the proportions were similar, though non-significant (74.1% vs 92.9%, p=0.06). There was a significant linear correlation between anti-N IgG and Omicron NAb titers (R^2^=0.27, p<0.0001), due to the contribution of very low and very high-titer anti-N IgG samples (Figure 3B).

Finally, we determined if viral shedding duration was linked to Omicron NAb titers. Comparing SARS-CoV-2 cases with a short duration of viral shedding (≤7 days) to longer duration (>7 days), there were no differences in median Omicron NAb titer overall (27.0 vs 54.1, n=0.61), or stratified by sex (p=0.60) or COVID-19 vaccination (p=0.98).

## DISCUSSION

We previously found a high SARS-CoV-2 force of infection in a cohort of Guatemalan agricultural workers (17, 18) and their households (19). Leveraging the same household cohort, we now evaluated the protective role of pre-existing SARS-CoV-2 antibodies (IgG binding and neutralization) against SARS-CoV-2 infection and shedding duration. We found that COVID-19 cases were more likely to have received fewer vaccine doses and were less likely to have preceding hybrid immunity. These findings are consistent with other global studies showing an incremental protective benefit of booster doses and staying up-to-date on vaccination, though the benefit waned quickly over 6 months and with the arrival of new subvariants (33–36). We previously showed rapid waning of NAb titers over 6 months in the AGRI farm worker cohort (18). Future studies should confirm if increased vaccine boosters promoted NAb durability against circulating and future variants in this cohort, as reported in other field sites (37, 38).

Importantly, we showed that both cumulative vaccine doses and recency was associated with higher NAb titers against the circulating strain. Among the 27 SARS-CoV-2 “breakthrough” cases who were considered up-to-date on vaccination yet were still infected, NAb titers were significantly lower than controls (13.1 vs 155.6), suggesting a failure of vaccine to induce an appropriate NAb response among these individuals. This finding has been reported elsewhere (39, 40), and should be explored further in future studies.

Our high nucleocapsid IgG seropositivity (18) and vaccination rate provided an opportunity to study hybrid immunity (41). Indeed, among vaccinated individuals, prior virus infection/exposure was associated with higher levels of NAb against the circulating strain (Omicron) and increased SARS-CoV-2 protection. Zhong *et al* had similar findings, noting in a pediatric cohort (5–12 years old) from Singapore that NAb titer only correlated with protection against symptomatic COVID-19 in the setting of hybrid immunity and that T cell immunity was the best correlate of protection prior to hybrid immunity (42). However, not all studies consistently support these findings (3–12).

We found that high Omicron NAb titers correlated with nucleocapsid IgG titers (anti-S titers were evaluated in a subset of individuals but were consistently above the limit of detection). Nucleocapsid IgG titers are technically more straightforward to measure than NAb titers, suggesting a potential biomarker for immunity among vaccinated individuals in resource-limited settings. As nucleocapsid is not present in the vaccine, we speculate that this relationship may be due to those with SARS-CoV-2 infections having lower preceding anti-nucleocapsid (infection-induced) immunity. In fact, the high magnitude and durability of NAb responses in the context of hybrid immunity were associated with the induction of memory B cells that in turn was associated with enhanced CD4 T cell responses (43). It would be of interest to understand the T cell responses against diverse viral antigens including nucleocapsid, and the nature of spike epitopes recognized by NAbs in this household cohort. Specifically, in-depth monitoring of household contacts in the AGRI-CASA cohort provides a rare opportunity to test the relationship between immune phenotypes and transmission parameters, such as the duration of viral shedding. Here, serum IgG and NAb titers did not predict the duration of viral shedding, with previous studies showing mixed associations, including a potential role for secretory IgA (44–47).

Strengths of our study included a high household enrollment requirement (>75%), frequent saliva sampling irrespective of presence of symptoms, timely surveying and testing of household infections prompted by symptomatic individuals, accurate vaccination records, and a team of experienced, well-trained field and laboratory staff in this rural community. One weakness was that we did not capture SARS-CoV-2 infection history prior to study enrollment, including timing of prior infections, which began in September 2021 when much of the community was already infected. Our SARS-CoV-2 serologic testing occurred up to 12 months prior to infection, which likely resulted in less precise measurements given the known decline in NAb titers over shorter intervals. Although we matched specimens on calendar time, an increased proportion of vaccinated control specimens collected during Omicron circulation could explain relatively higher NAb titers in this group due to exposures; however, there were no difference in NAb titers when we compared unvaccinated cases and controls during this same time period. We also did not evaluate other correlates of protection, such as memory T cell response, which likely play a significant role that may increase over time.

## CONCLUSIONS

In a cohort from Guatemala we found that cumulative COVID-19 vaccine doses and greater anti-N IgG and Omicron NAb titer were associated with lower odds of SARS-CoV-2 infection, suggesting a protective role for hybrid immunity. These findings support the importance of continued vaccination efforts in Guatemala, particularly among essential worker populations and their household members who are at high risk of infection and disease. Given a high prevalence of natural infection, and rapid waning of vaccine-induced antibodies, continued vaccination will likely provide the best approach to prevent COVID-19 disease in this population, which we have demonstrated results in significant clinical and economic impacts (48). Nucleocapsid IgG titers may serve as a useful correlate of protection among individuals with hybrid immunity. Future studies should focus on mucosal and systemic longitudinal immunity in the prevention of SARS-CoV-2 infection and disease, including with next generation vaccines.

## Supplementary Material

Supplementary Files

This is a list of supplementary files associated with this preprint. Click to download.

- SupplementalTablesandFigures9apr2026a.docx

## Figures and Tables

**Table 1:** Comparison of demographics between SARS-CoV-2 cases and matched uninfected noncases (controls).

Demographic Variable	All subjects	SARS-CoV-2 Cases	Controls	p-value (cases vs controls)
	N = 220	N = 119	N = 101	
**Age in years**	23.0 (16.5)	22.7 (15.6)	23.3 (17.5)	0.78
**Sex = male**	99 (45.0%)	47 (39.5%)	52 (51.5%)	0.08
**Status**				
** Index or Co-index**	55 (25.0%)	55 (46.2%)	0 (0%)	
** Secondary**	56 (25.5%)	56 (47.1%)	0 (0%)	N/A
** Tertiary**	8 (3.6%)	8 (6.7%)	0 (0%)	
** Not a case**	101 (45.9%)	0 (0%)	101 (100%)	
**Pre-existing medical condition**	35 (15.9%)	19 (16.0%)	16 (15.8%)	0.98
**# vaccine doses at time of enrollment (sample collection date):** *				
** 0**	142 (64.55%)	81 (68.1%)	61 (60.4%)	0.07
** 1**	20 (9.09%)	11 (9.24%)	9 (8.9%)	
** 2**	41 (18.64%)	23 (19.33%)	18 (17.82%)	
** 3**	17 (7.73%%)	4 (3.36%)	13 (12.87%)	
**Received a vaccine dose during the past 12 months (if completed primary series)**	58 (26.6%)	27 (22.7%)	31 (30.7%)	0.18
**Hybrid Immunity status at sample collection date** **				
** Unvaccinated/No prior infection**	35 (16.0%)	17 (14.3%)	18 (18.0%)	
** Vaccinated/No prior infection**	15 (6.8%)	11 (9.2%)	4 (4.0%)	0.08
** Unvaccinated/Prior Infection**	111 (50.7%)	66 (55.5%)	45 (45.0%)	
** Vaccinated/Prior infection (hybrid)**	58 (26.5%)	25 (21.0%)	33 (33.0%)	

* Cases were enrolled at the time of first confirmed SARS-CoV-2 infection; for controls, we used the same date as their matched cases. Six controls, which were matched based on the date of serum collection, were excluded because they had ended study participation by the time their matched cases developed SARS-CoV-2 infection.

** Prior infection was inferred from nucleocapsid IgG positivity and hybrid immunity was defined based on having both documented vaccination and prior infection. Note that one vaccinated control had no nucleocapsid IgG data due to sample availability.

**Table 2:** Comparison of SARS-CoV-2 IC50 values preceding SARS-CoV-2 infection (cases) and in matched non-infected controls. (Wilcoxon test by sex and by vaccination status)

Demographic Variable	SARS-CoV-2 Cases N = 119	Controls N = 101	p-value
	Median (IQR)	Median (IQR)	
**Overall**	28.4 (2.0, 168.2)	50.4 (12.5, 215.9)	0.15
**Male sex**	19.0 (2.0, 114.2)	43.7 (10.3, 215.9)	0.16
**# vaccine doses at enrollment (sample collection date:** *			
** 0**	38.9 (9.2, 270.2)	24.5 (2.0, 68.4)	0.11
** 1**	16.1 (2.0, 68.9)	182.7 (45.2, 245.3)	0.01
** 2**	11.9 (2.0, 108.5)	87.0 (31.3, 325.5)	0.04
** 3**	13.4 (7.6, 16.4)	232.5 (179.6, 507.0)	0.004
**Considered “up-to-date” at the time of enrollment (sample collection date)**	13.1 (2.0, 58.5)	155.6 (79.2, 447.8)	0.0001
**Hybrid Immunity status at sample collection date** **			
** Unvaccinated/No prior infection**	2.0 (2.0, 74.0)	2.0 (2.0, 14.2)	0.65
** Vaccinated/No prior infection**	2.0 (2.0, 11.3)	78.8 (2.0, 185.75)	0.44
** Unvaccinated/Prior Infection**	59.2 (12.2, 347.0)	41.8 (14.6, 153.7)	0.34
** Vaccinated/Prior infection (hybrid)**	24.6 (11.9, 91.1)	184.0 (87.4, 491.7)	<0.0001

* Vaccinated prior to the time of serum sample collection.

** includes only 4 cases and 13 controls

## LIST OF ABBREVIATIONS

AGRI-CASA: Agricultural Worker COVID-19 Asymptomatic and Symptomatic Transmission in the Home and Workplace Study
CLI: COVID-19-like illness
COI: cut-off index
NAb: neutralizing antibody
CU-AMC: University of Colorado Anschutz Medical Campus; IC50, 50% inhibitory conc
IQR: interquartile range
POC: point-of-care
RT-PCR: reverse transcriptase polymerase chain reaction

## References

[1] Chicas R, Xiuhtecutli N, Houser M, Glastra S, Elon L, Sands JM, COVID-19 and Agricultural Workers: A Descriptive Study. J Immigr Minor Health. 2022 Feb;24(1):58–64. 34637039 10.1007/s10903-021-01290-9 PMC8507360

[2] Miller JS, Holshue M, Dostal TKH, Newman LP, Lindquist S. COVID-19 Outbreak Among Farmworkers - Okanogan County, Washington, May-August 2020. MMWR Morb Mortal Wkly Rep. 2021 Apr 30;70(17):617–21. 33914719 10.15585/mmwr.mm7017a3 PMC8084124

[3] Earle KA, Ambrosino DM, Fiore-Gartland A, Goldblatt D, Gilbert PB, Siber GR, Evidence for antibody as a protective correlate for COVID-19 vaccines. Vaccine. 2021 Jul 22;39(32):4423–8. 34210573 10.1016/j.vaccine.2021.05.063 PMC8142841

[4] Feng S, Phillips DJ, White T, Sayal H, Aley PK, Bibi S, Correlates of protection against symptomatic and asymptomatic SARS-CoV-2 infection. Nat Med. 2021 Nov;27(11):2032–40. 34588689 10.1038/s41591-021-01540-1 PMC8604724

[5] Goldblatt D, Alter G, Crotty S, Plotkin SA. Correlates of protection against SARS-CoV-2 infection and COVID-19 disease. Immunol Rev. 2022 Sep;310(1):6–26. 35661178 10.1111/imr.13091 PMC9348242

[6] Goldblatt D, Fiore-Gartland A, Johnson M, Hunt A, Bengt C, Zavadska D, Towards a population-based threshold of protection for COVID-19 vaccines. Vaccine. 2022 Jan 21;40(2):306–15. 34933765 10.1016/j.vaccine.2021.12.006 PMC8673730

[7] Khoury DS, Cromer D, Reynaldi A, Schlub TE, Wheatley AK, Juno JA, Neutralizing antibody levels are highly predictive of immune protection from symptomatic SARS-CoV-2 infection. Nat Med. 2021 Jul;27(7):1205–11. 34002089 10.1038/s41591-021-01377-8

[8] Khoury DS, Docken SS, Subbarao K, Kent SJ, Davenport MP, Cromer D. Predicting the efficacy of variant-modified COVID-19 vaccine boosters. Nat Med. 2023 Mar;29(3):574–8. 36864253 10.1038/s41591-023-02228-4

[9] Khoury DS, Schlub TE, Cromer D, Steain M, Fong Y, Gilbert PB, Correlates of Protection, Thresholds of Protection, and Immunobridging among Persons with SARS-CoV-2 Infection. Emerg Infect Dis. 2023 Feb;29(2):381–8. 36692375 10.3201/eid2902.221422 PMC9881762

[10] Regev-Yochay G, Lustig Y, Joseph G, Gilboa M, Barda N, Gens I, Correlates of protection against COVID-19 infection and intensity of symptomatic disease in vaccinated individuals exposed to SARS-CoV-2 in households in Israel (ICoFS): a prospective cohort study. Lancet Microbe. 2023 May;4(5):e309–e18. 36963419 10.1016/S2666-5247(23)00012-5 PMC10030121

[11] Wei J, Matthews PC, Stoesser N, Newton JN, Diamond I, Studley R, Protection against SARS-CoV-2 Omicron BA.4/5 variant following booster vaccination or breakthrough infection in the UK. Nat Commun. 2023 May 16;14(1):2799. 37193713 10.1038/s41467-023-38275-1 PMC10187514

[12] Wei J, Pouwels KB, Stoesser N, Matthews PC, Diamond I, Studley R, Antibody responses and correlates of protection in the general population after two doses of the ChAdOx1 or BNT162b2 vaccines. Nat Med. 2022 May;28(5):1072–82. 35165453 10.1038/s41591-022-01721-6 PMC9117148

[13] Chen YH, Glymour M, Riley A, Balmes J, Duchowny K, Harrison R, Excess mortality associated with the COVID-19 pandemic among Californians 18–65 years of age, by occupational sector and occupation: March through November 2020. PLoS One. 2021;16(6):e0252454. 34086762 10.1371/journal.pone.0252454 PMC8177528

[14] Lusk JL, Chandra R. Farmer and farm worker illnesses and deaths from COVID-19 and impacts on agricultural output. PLoS One. 2021;16(4):e0250621. 33909685 10.1371/journal.pone.0250621 PMC8081247

[15] Lewnard JA, Mora AM, Nkwocha O, Kogut K, Rauch SA, Morga N, Prevalence and Clinical Profile of Severe Acute Respiratory Syndrome Coronavirus 2 Infection among Farmworkers, California, USA, June-November 2020. Emerg Infect Dis. 2021 May;27(5):1330–42. 33657340 10.3201/eid2705.204949 PMC8084509

[16] Follmann D, O’Brien MP, Fintzi J, Fay MP, Montefiori D, Mateja A, Examining protective effects of SARS-CoV-2 neutralizing antibodies after vaccination or monoclonal antibody administration. Nat Commun. 2023 Jun 17;14(1):3605. 37330602 10.1038/s41467-023-39292-w PMC10276829

[17] Olson D, Calvimontes DM, Lamb MM, Guzman G, Barrios E, Chacon A, Clinical and Economic Impact of COVID-19 on Plantation Workers: Preliminary Results from the Guatemala Agricultural Workers and Respiratory Illness Impact (AGRI) Study. medRxiv. 2022 Feb 8.

[18] Iwamoto C, Lesteberg KE, Lamb MM, Calvimontes DM, Guo K, Barrett BS, High SARS-CoV-2 Seroprevalence and Rapid Neutralizing Antibody Decline among Agricultural Workers in Rural Guatemala, June 2020-March 2021. Vaccines (Basel). 2022 Jul 21;10(7). 10.3390/vaccines10071160 PMC9323551 35891324

[19] Carreon JD, Lamb MM, Chard AN, Calvimontes DM, Iwamoto C, Rojop N, SARS-CoV-2 secondary attack rates and risks for transmission among agricultural workers and their households in Guatemala, 2022–2023. IJID Reg. 2025 Sep;16:100676. 40606590 10.1016/j.ijregi.2025.100676 PMC12210297

[20] Asturias EJ, Heinrichs G, Domek G, Brett J, Shick E, Cunningham M, The Center for Human Development in Guatemala: An Innovative Model for Global Population Health. Adv Pediatr. 2016 Aug;63(1):357–87. 27426907 10.1016/j.yapd.2016.04.001

[21] Olson D, Lamb M, Lopez MR, Colborn K, Paniagua-Avila A, Zacarias A, Performance of a Mobile Phone App-Based Participatory Syndromic Surveillance System for Acute Febrile Illness and Acute Gastroenteritis in Rural Guatemala. J Med Internet Res. 2017 Nov 9;19(11):e368. 29122738 10.2196/jmir.8041 PMC5701088

[22] National Center for Immunization and Respiratory Diseases (NCIRD) DoVD. Symptoms of COVID-19. Mar. 15, 2024 [cited 2024 2024]; Available from: https://www.cdc.gov/coronavirus/2019-ncov/symptoms-testing/symptoms.html

[23] Reses HE, Fajans M, Lee SH, Heilig CM, Chu VT, Thornburg NJ, Performance of existing and novel surveillance case definitions for COVID-19 in household contacts of PCR-confirmed COVID-19. BMC Public Health. 2021 Sep 25;21(1):1747. 34563163 10.1186/s12889-021-11683-y PMC8465785

[24] Vogels CBF, Watkins AE, Harden CA, Brackney DE, Shafer J, Wang J, SalivaDirect: A simplified and flexible platform to enhance SARS-CoV-2 testing capacity. Med. 2021 Mar 12;2(3):263–80.e6. 33521748 10.1016/j.medj.2020.12.010 PMC7836249

[25] Paiz-Reyes C, Arias K, Del Cid-Villatoro J, Vasquez D, Gomez M, Rojop N, Comparison between Saliva and Nasopharyngeal Swabs for the Rapid Detection of Respiratory Viruses Using the Roche Cobas(R) Liat(R) Polymerase Chain Reaction System in Rural Guatemala. Am J Trop Med Hyg. 2025 May 27. 10.4269/ajtmh.24-0184 PMC12360069 40424997

[26] Koski RR, Klepser ME. A systematic review of rapid diagnostic tests for influenza: considerations for the community pharmacist. J Am Pharm Assoc (2003). 2017 Jan-Feb;57(1):13–9. 27836481 10.1016/j.japh.2016.08.018

[27] Shu B, Kirby MK, Davis WG, Warnes C, Liddell J, Liu J, Multiplex Real-Time Reverse Transcription PCR for Influenza A Virus, Influenza B Virus, and Severe Acute Respiratory Syndrome Coronavirus 2. Emerg Infect Dis. 2021;27(7):1821–30. 34152951 10.3201/eid2707.210462 PMC8237866

[28] Wagner K, Fox P, Gordon E, Hahn W, Olsen K, Markham A, A multiplexed, paired-pooled droplet digital PCR assay for detection of SARS-CoV-2 in saliva. Sci Rep. 2023 Feb 22;13(1):3075. 36813822 10.1038/s41598-023-29858-5 PMC9944410

[29] Muench P, Jochum S, Wenderoth V, Ofenloch-Haehnle B, Hombach M, Strobl M, Development and Validation of the Elecsys Anti-SARS-CoV-2 Immunoassay as a Highly Specific Tool for Determining Past Exposure to SARS-CoV-2. J Clin Microbiol. 2020 Sep 22;58(10). 10.1128/JCM.01694-20 PMC7512151 32747400

[30] Hasenkrug KJ, Feldmann F, Myers L, Santiago ML, Guo K, Barrett BS, Recovery from Acute SARS-CoV-2 Infection and Development of Anamnestic Immune Responses in T Cell-Depleted Rhesus Macaques. mBio. 2021 Aug 31;12(4):e0150321. 34311582 10.1128/mBio.01503-21 PMC8406331

[31] Zhou P, Yang XL, Wang XG, Hu B, Zhang L, Zhang W, A pneumonia outbreak associated with a new coronavirus of probable bat origin. Nature. 2020 Mar;579(7798):270–3. 32015507 10.1038/s41586-020-2012-7 PMC7095418

[32] Guo K, Barrett BS, Mickens KL, Vladar EK, Morrison JH, Hasenkrug KJ, Interferon Resistance of Emerging SARS-CoV-2 Variants. bioRxiv. 2021 Dec 10. 10.1073/pnas.2203760119 PMC9371743 35867811

[33] VIEW-hub. [cited 2025 6/23/2025]; Available from: https://view-hub.org/vaccine/covid/neutralization-studies

[34] Feikin DR, Higdon MM, Abu-Raddad LJ, Andrews N, Araos R, Goldberg Y, Duration of effectiveness of vaccines against SARS-CoV-2 infection and COVID-19 disease: results of a systematic review and meta-regression. Lancet. 2022 Mar 5;399(10328):924–44. 35202601 10.1016/S0140-6736(22)00152-0 PMC8863502

[35] Chemaitelly H, Ayoub HH, Tang P, Coyle P, Yassine HM, Al Thani AA, Long-term COVID-19 booster effectiveness by infection history and clinical vulnerability and immune imprinting: a retrospective population-based cohort study. Lancet Infect Dis. 2023 Jul;23(7):816–27. 36913963 10.1016/S1473-3099(23)00058-0 PMC10079373

[36] Lee N, Nguyen L, Austin PC, Brown KA, Grewal R, Buchan SA, Protection Conferred by COVID-19 Vaccination, Prior SARS-CoV-2 Infection, or Hybrid Immunity Against Omicron-Associated Severe Outcomes Among Community-Dwelling Adults. Clin Infect Dis. 2024 May 15;78(5):1372–82. 38001037 10.1093/cid/ciad716 PMC11093681

[37] Korosec CS, Dick DW, Moyles IR, Watmough J. SARS-CoV-2 booster vaccine dose significantly extends humoral immune response half-life beyond the primary series. Sci Rep. 2024 Apr 18;14(1):8426. 38637521 10.1038/s41598-024-58811-3 PMC11026522

[38] Sanada T, Honda T, Kohara M. Modeling of anti-spike IgG and neutralizing antibody waning after anti-SARS-CoV-2 mRNA vaccination. Vaccine. 2024 Aug 30;42(21):126146. 39033078 10.1016/j.vaccine.2024.07.047

[39] Bergwerk M, Gonen T, Lustig Y, Amit S, Lipsitch M, Cohen C, Covid-19 Breakthrough Infections in Vaccinated Health Care Workers. N Engl J Med. 2021 Oct 14;385(16):1474–84. 34320281 10.1056/NEJMoa2109072 PMC8362591

[40] Lake DF, Roeder AJ, Gonzalez-Moa MJ, Koehler M, Kaleta E, Jasbi P, Third COVID-19 vaccine dose boosts neutralizing antibodies in poor responders. Commun Med (Lond). 2022;2:85. 35832309 10.1038/s43856-022-00151-2 PMC9273613

[41] Bobrovitz N, Ware H, Ma X, Li Z, Hosseini R, Cao C, Protective effectiveness of previous SARS-CoV-2 infection and hybrid immunity against the omicron variant and severe disease: a systematic review and meta-regression. Lancet Infect Dis. 2023 May;23(5):556–67. 36681084 10.1016/S1473-3099(22)00801-5 PMC10014083

[42] Zhong Y, Kang AYH, Tay CJX, Li HE, Elyana N, Tan CW, Correlates of protection against symptomatic SARS-CoV-2 in vaccinated children. Nat Med. 2024 May;30(5):1373–83. 38689059 10.1038/s41591-024-02962-3 PMC11164684

[43] Crotty S. Hybrid Immunity. Science. 2021 25 june, 2021;372(6549):1392–3.

[44] Miyamoto S, Nishiyama T, Ueno A, Park H, Kanno T, Nakamura N, Infectious virus shedding duration reflects secretory IgA antibody response latency after SARS-CoV-2 infection. Proc Natl Acad Sci U S A. 2023 Dec 26;120(52):e2314808120. 38134196 10.1073/pnas.2314808120 PMC10756199

[45] Yang Y, Guo L, Yuan J, Xu Z, Gu Y, Zhang J, Viral and antibody dynamics of acute infection with SARS-CoV-2 omicron variant (B.1.1.529): a prospective cohort study from Shenzhen, China. Lancet Microbe. 2023 Aug;4(8):e632–e41. 37459867 10.1016/S2666-5247(23)00139-8

[46] Maier HE, Plazaola M, Lopez R, Sanchez N, Saborio S, Ojeda S, SARS-CoV-2 infection-induced immunity and the duration of viral shedding: Results from a Nicaraguan household cohort study. Influenza Other Respir Viruses. 2023 Jan;17(1):e13074. 36457275 10.1111/irv.13074 PMC9835439

[47] Puhach O, Meyer B, Eckerle I. SARS-CoV-2 viral load and shedding kinetics. Nat Rev Microbiol. 2023 Mar;21(3):147–61. 36460930 10.1038/s41579-022-00822-w PMC9716513

[48] Olson D, Calvimontes DM, Lamb MM, Guzman G, Barrios E, Chacon A, Clinical and Economic Impact of COVID-19 on Agricultural Workers, Guatemala(1). Emerg Infect Dis. 2022 Dec;28(13):S277–S87. 36502430 10.3201/eid2813.212303 PMC9745239

